# Evaluating the causal association between resting state intrinsic functional networks and the risk for neurodegeneration

**DOI:** 10.1002/alz.094993

**Published:** 2025-01-09

**Authors:** Malik Nassan, Iyas Daghlas, Bram R Diamond, Adam Martersteck, Emily J Rogalski

**Affiliations:** ^1^ Mesulam Center for Cognitive Neurology, Northwestern University Feinberg School of Medicine, Chicago, IL USA; ^2^ Department of Neurology, University of California San Francisco, San Francisco, CA USA; ^3^ University of Chicago, Chicago, IL USA; ^4^ Northwestern University Feinberg School of Medicine, Chicago, IL USA

## Abstract

**Background:**

Alterations of resting state intrinsic functional networks have been associated with neurodegenerative diseases even before the onset of cognitive symptoms. Emerging hypotheses propose a role of intrinsic functional networks alterations in the risk or vulnerability to neurodegeneration. It is unknown whether intrinsic functional network alterations can be causal for neurodegenerative diseases. We sought to answer this question using two‐sample Mendelian randomization, a method able to assess causal relationships.

**Methods:**

Using the largest genome‐wide association study of resting state intrinsic functional connectivity (n = 47,276), we generated genetic instruments (at the significance level 2.8 × 10^−11^) to proxy resting state intrinsic functional network features. From these, we selected 26 genetically proxied resting state intrinsic functional features from brain regions implicated in neurodegenerative diseases. We tested their association with the paired neurodegenerative outcomes: Alzheimer dementia (111,326 cases, 677,663 controls), frontotemporal dementia (2,154 cases, 4,308 controls), semantic dementia (308 cases, 616 controls), and Lewy body dementia (2,591 cases, 4,027 controls). Major depressive disorder outcome (170,756 cases, 329,443 controls) was included as a positive control. Power and sensitivity analyses were completed to assess the robustness of the results.

**Results:**

None of the genetically proxied resting state intrinsic functional network features were significantly associated with neurodegenerative disease outcomes (adjusted P value >0.05), despite sufficient power. However, two resting state exposures in the visual cortex showed a nominal level of association with Lewy body dementia (P = 0.01), a finding that was supported using a separate visual cortex connectivity exposure (P = 0.03). For our confirmatory positive control, the global connectivity pattern in the default mode network was associated with risk of major depressive disorder (P = 0.024), supporting the validity of the selected genetic instruments. Sensitivity analyses were supportive of the main results.

**Conclusion:**

This is the first study to comprehensively assess the potential causal effect of resting state intrinsic functional network features on risk of neurodegenerative diseases. Overall, the results do not support a causal role for intrinsic functional network connectivity in the pathogenesis of neurodegenerative disorders. However, the suggestive association between visual network connectivity and risk of Lewy body dementia requires further evaluation.

